# Temporal dynamics: A systematic review and meta-analysis of time intervals in time-based prospective memory and their connection to time perception

**DOI:** 10.3758/s13423-025-02822-2

**Published:** 2026-02-18

**Authors:** Farkhondeh Fakour Manavi, Paul D. Loprinzi, Rebekah E. Smith

**Affiliations:** 1 Department of Psychology, University of Mississippi, University, MS 38677 USA; 2 Department of Exercise Science, University of Mississippi, University, MS USA

**Keywords:** Time-based prospective memory, Time interval, Delay interval, Time perception

## Abstract

Time-based prospective memory refers to the ability to remember activities at a specified future time. In our everyday lives, certain time-based tasks need to be completed within a short time period, while others are meant for a more distant future. We report the findings from two separate but related reviews regarding the temporal dynamics of time-based prospective memory. The first included a systematic and meta-analytic review of the impact of short versus long time intervals on the time-based prospective memory performance. Ninety-three comparisons were included in the first meta-analysis, revealing a medium effect size associated with time intervals on time-based prospective memory performance, indicating superior performance with shorter delay intervals. The second section of this report comprises a review and meta-analysis of the correlation between time-based prospective memory and individuals’ perception of time. The findings from 18 comparisons in the second part of the study suggest a negative correlation between time-based prospective memory and time perception. In summary, the results of these reviews provide evidence of the influence of short and long time intervals on time-based prospective memory performance and highlights the connection between time-based prospective memory performance and individuals’ time perception. The findings, which are considered in relation to theoretical explanations of time-based prospective memory, point to several avenues for future study.

## Introduction

Prospective memory (PM) is memory for future intentions, specifically, PM tasks involve having an intention to carry out an action when that action cannot be completed immediately and instead must be delayed. Prospective memory tasks can be classified into different types (Kvavilashvili & Ellis, 1996) with two commonly used classifications, time-based PM and event-based PM. In event-based PM, the planned action is to be performed in response to an event in the environment, such as remembering to return your book to the library when you see the library on campus. On the other hand, activities could be planned to be performed at a particular time-point or after a period of time has elapsed in time-based PM tasks. Examples of time-based PM include calling your friend at 3 p.m. or taking your medication in an hour (Einstein & McDaniel, 1990, 1996). Successful everyday functioning depends on completing these delayed intention tasks. Taking medications, watching your favorite television show, attending your classes, remembering your loved ones’ birthday, or paying your bills are just some examples of time-based activities in our medical, personal, and social life. Time-based PM tasks are the focus of this review.

### Aims of this review

Although time-based PM tasks mostly have fixed and repetitive intervals in laboratory paradigms (e.g., press a key every 2 min), PM tasks in a real life have different time intervals from a few minutes (e.g., a 15-min time interval from the moment you put the food in the oven until you take it out of it) to several days (e.g., call your friends the next weekend to invite them for your party) or even weeks (e.g., remembering the exact time of your next month bill). Despite numerous examples of different time intervals in our life, the length of the target time interval has surprisingly been largely overlooked as a variable of interest in PM research. To our knowledge, a few published papers manipulated time interval as a main independent variable to investigate the effects of varying time intervals in time-based PM. Thus, a broad aim of the present review paper is to provide an overview of the existing studies that have compared short versus long time intervals in time-based PM tasks.

In completing a time-based task in real life, we do not check the clock constantly until the target point approaches, but rather monitor the clock periodically and estimate the passing time internally to keep track of time. For example, if you have an appointment at 11 a.m., you do not look at your watch every minute in the morning, but instead check it periodically and estimate the passage of time in between checks to follow time progression. Prior research has indicated that individuals tend to monitor the clock more frequently as the target time approaches, rather than consistently checking it throughout the task. This behavior has been associated with improved performance in time-based PM performance (Jägar & Kliegel, 2008; Maylor et al., 2002). This raises the question of how individuals estimate the passage of time between these instances of checking an external clock. One possible explanation is that they internally estimate the elapsed time based on their anticipation of how long their ongoing or upcoming activities will take. For instance, if it is currently 9 a.m. and you have a meeting at 11 a.m., you might decide to spend this time reading a paper. Depending on the number of pages in the paper and your reading speed, you may estimate that reading it will require 90 min, leaving you with 30 min to prepare before your meeting. Consequently, while engaged in reading, you might not feel the need to constantly check the clock. In connection with this, a prior study showed a positive relationship between time management and the ability to estimate the duration of current or future activities in healthy older adults (Francis-Smythe & Robertson, 1999). The ability to estimate and compare the duration of everyday activities has also been investigated in another study in children with disabilities (Janeslätt et al., 2009). The study examined time-processing ability (e.g., time perception, time orientation, and time management) in children with a disability (e.g., attention-deficit/hyperactivity disorder, autism, or physical or intellectual disabilities) and demonstrated a correlation between children’s time-processing ability and their time management as rated by their parents. These studies represent the multifaceted nature of time-related cognitive abilities, spanning from internal time estimation and external clock monitoring to the ability to effectively manage time in daily activities. By considering these diverse perspectives, we can gain a more comprehensive understanding of time-based tasks. Therefore, the second goal of the present study is to review previous work regarding the role of time perception in time-based PM.

The final aims of this review will be to identify the limitations of the existing studies, to point out the possible ways to address these limitations and to suggest avenues for future research. Before reviewing the empirical work on the questions of time intervals and time perception, we first introduce basic methodological approaches to studying time-based PM and discuss the earliest explanatory model of how we perform time-based PM tasks.

### A brief look at time-based prospective memory (PM) paradigms

#### The ongoing task

One of the important facets of PM tasks is that these tasks are performed while one is engaged in other activities (McDaniel & Einstein, 2007). For example, you might review your calendar in the morning and see that you have an appointment at 1 p.m., but before being able to complete the PM task of going to the appointment, you have to engage in other activities such as making breakfast, taking children to school, and checking your emails. These other activities occupy your attentional resources and at the same time you need to check the clock periodically to perform the PM task at the appropriate time.

For studying PM in a laboratory setting, the PM task is embedded in an ongoing task, which is usually a cognitive task, such as a working memory or a lexical decision task, to keep participants’ attentional resources well engaged. For example, in a study, a series of letters appeared on the screen, with the letters in the first, third, and fifth positions consistently being identical. However, the letters in the second and fourth positions could be either the same (e.g., DFDFD) or different (e.g., DFDGD). Participants were required to press one key if the letters in the second and fourth positions matched and another key if they were different. Additionally, participants were instructed to press a key every 5 min throughout the task (Cona et al., 2012).

In addition, using a cognitive task as an ongoing task makes it feasible to measure the possible detrimental effects of adding a PM task to the ongoing task by comparing the accuracy and reaction times of the ongoing task for participants who perform the PM task with ongoing task performance when the ongoing task is performed without the added PM task. Changes in ongoing task performance can serve as an indicator of the resource demands associated with the addition of the PM task. Smith (2003) first highlighted^1^ this use of the ongoing task to evaluate the resource demands on event-based PM tasks. Subsequently, it has been repeatedly shown in the literature that the addition of a time-based PM task also results in a performance cost to the ongoing tasks, typically with the finding that reaction time of the ongoing task slows down when a PM task is added to the ongoing task (Hicks et al., 2005). Thus, in addition to reviewing the existing findings on how time interval length affects time-based PM performance (i.e., the completion of the PM task itself), we will review how interval length affects ongoing task performance.

#### Clock checking

One of the factors contributing to successful accomplishment of time-based PM tasks is time monitoring (Mioni & Stablum, 2014). In other words, to find the appropriate time to respond, one must monitor the progression of time and periodically check the clock. For example, to make sure not to forget to call your friend at 9 p.m., you must check the clock regularly to monitor the passage of time while you are watching TV, cooking dinner, or attending other activities.

Harris and Wilkins (1982) designed a study to examine how individuals engage in time monitoring when they are required to carry out delayed intentions with different time intervals. In their study, participants were asked to hold up a series of cards at specified target times (i.e., 3 or 9 min) while they were watching a movie. This study demonstrated an important result: participants checked the clock more frequently as they got closer to the target point. Based on this finding, Harris and Wilkins (1982) proposed the Test-Wait-Test-Exit (TWTE) model. According to this model, in the test phase, people evaluate the environment to check whether the target time to perform the delayed intention is close. If participants are not close enough to the target time, they wait for a period of time. Then they test again, and the procedure continues until a test indicates it is the proper time to respond. At the target time, they perform the action and exit the loop.

Additional studies have also indicated that successful clock checking presents in a J-shaped function; during the first part of the time-based PM task, the clock is checked less frequently, and clock checking increases closer to the target time to guarantee a successful time-based PM performance (Kliegel et al., 2001; Mäntylä & Carelli, 2006). Research has further demonstrated that it is not the overall clock-checking frequency, but strategic clock checking that occurs around the target time that predicts time-based PM (Ceci & Bronfenbrenner, 1985; Zinke et al., 2010).

In addition, the study by Harris and Wilkins showed that the frequency of clock checking was not correlated with short versus long intervals. Although Harris and Wilkins examined different time intervals in their study, further studies have primarily replicated their findings regarding time monitoring with fixed time intervals and the question of how different time intervals affect clock checking is also relatively neglected in PM literature. Thus, our overview also summarizes studies that have examined the effects of time interval variability on clock-checking behavior.

### Overview

The remainder of this paper is presented in three sections. Review 1 presents a review of empirical findings in experiments comparing short versus long time intervals in time-based PM. Review 2 summarizes findings regarding the relationship between time-perception and time-based PM. In the final concluding section, the results of these two separate reviews will be considered together as we cover limitations of the existing work, and we discuss avenues for future research.

## Review 1: Summary of empirical findings of time interval studies

As discussed in the *Introduction*, time-based PM tasks in our daily lives cover a range of time intervals. Despite the rapid expansion of work in PM over the last 30 years, our literature review produced few studies that have systematically investigated the effects of time-interval manipulations on time-based PM. This first review covers three dependent variables that are frequently included in time-based PM tasks: PM task performance itself, clock checking, and ongoing task performance in separate sections. With respect to the first dependent measure, we also include a short overview of the influence of time intervals on PM performance of healthy and clinical populations, and an analysis of effect sizes.

Although the majority of previous studies have compared PM performance between healthy and clinical populations (see meta-analysis by Román Caballero & Mioni, 2023, for a review), the primary aim of the current meta-analysis was not to compare these groups but rather to examine time-based PM performance across short and long intervals. Therefore, we analyzed the effect sizes of both healthy and clinical populations together.

### Methods

Two authors (FFM and PDL) conducted literature searches independently, and a comparison was undertaken to ascertain the count of eligible studies. Agreement was achieved through consensus between these two reviewers. We reviewed published studies in three electronic databases: PsycINFO, PubMed, and Scopus from inception to December 2024. The following keywords were used to find relevant articles: “time-based prospective memory” OR “time based prospective memory” OR “time-based PM” OR “time based PM” AND “time interval” OR “time intervals” OR “delay interval” OR “delay intervals” OR “delay” OR “delays” OR “Memory for Intentions Screening Test” OR “Memory for Intentions Test” OR “MIST” OR “Royal Prince Alfred Prospective Memory Test” OR “RPA-ProMem.” We excluded review papers, meta-analyses, and book chapters from our search. Studies must have examined at least two different time intervals and report its effect on either time-based PM performance or time monitoring to be included. Neither participants’ age nor their mental or physical health was a factor to include or exclude studies. The only exclusion criterion was to exclude studies that investigated only event-based PM.

### Results

**Fig. 1:**
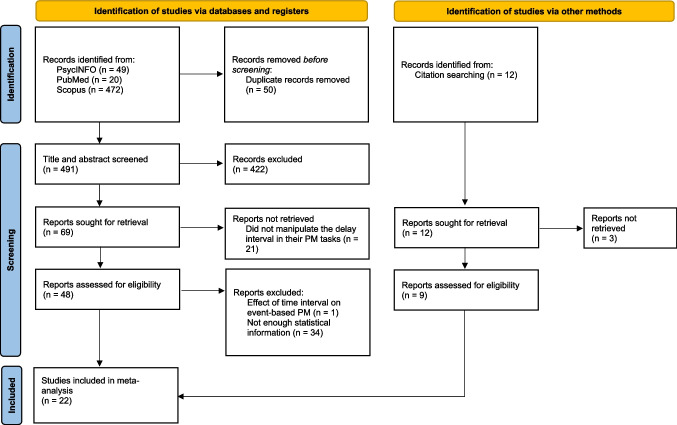
Flow chart showing article retrieval

Figure 1 illustrates the process of retrieving articles. A total of 541 relevant studies were identified, comprising 49 from PsycINFO, 20 from PubMed, and 472 from Scopus. After eliminating duplicate studies, the total was reduced to 491. Based on their titles and abstracts, 69 studies were initially selected, and after reviewing the full texts, 48 papers met the inclusion criteria for the review. Among these studies, 34 were excluded from the meta-analysis due to insufficient information for effect size calculation. In addition, one study was excluded because it examined the effect of delay on only event-based PM. Among the studies found by other sources, three were excluded due to a lack of sufficient statistical information. Thus, a total of 22 studies met the criteria and were considered eligible for meta-analysis. We used EndNote software (version 21) for duplicate checking and further investigation.

Table 1 presents the studies that have examined the effects of varying time intervals among healthy individuals and Table 2 presents the studies that have focused on clinical populations. As can be seen in Tables 1 and 2, previous studies evaluating the effects of varying time intervals have shown a range of results. We begin by describing the findings regarding PM performance, followed by a review of findings on clock checking and ongoing task performance.

**Table 1:** Description of studies investigating different time intervals in time-based prospective memory (PM) tasks in healthy individuals

Study	Age range (average age), years	Type of time-based PM task	Time intervals	Ongoing task	Effect of time intervals on PM
Bastin and Meulemans, 2002	Young adults: 20–30 Older adults: 60–70	Laboratory	1–2 min	Word recall	Yes
Black & McBride, 2024	Undergrad students	Naturalistic	1, 3, 6 days	Daily activities	Yes
Cantrelle et al., 2020	18–25	Both	20 min 24 h	- Daily activities	Yes^a^
Conte & McBride, 2018	Undergrad students	Laboratory	1, 3, 6 min	Lexical decision	No
D’Souza et al., 2024	18–25	Laboratory	1–15 min	Virtual shopping mall	N.R.
Green & Loprinzi, 2019	(Exercise: 20.4) (Control: 20.5)	Both	20 min 1 week	- Daily activities	Yes
Jie et al., 2016^a^	–	Laboratory	2–15 min	-	Yes
Kourtesis et al., 2021	Adults 18–45	Virtual Reality	15–30 min 45–60 min	Shopping, cooking in virtual environment	Yes
McBride et al., 2013	Young adults: 18–25 Older adults: 57–98	Naturalistic	1–2-5–14-28 days	Daily activities	Yes^b^
Nigro et al., 2002^a^	Children: 7–11	Laboratory	5–10 min	Mathematical operations and puzzles	Yes
Park et al., 1997	Young adults: 19.59 Older adults: 69.8	Laboratory	1–2 min	Word recall	Yes
Schnitzspahn et al., 2020	Young adults: 20–29 Older adults: 60–75	Naturalistic	1–3 days	Daily activities	Yes^c^
Stone et al., 2001^**d**^	Adults 18–40	Laboratory	1, 3, 5 min	Computer game	No
Tsai & Gilbert, 2024	Experiment 1: 26.4 Experiment 2: 27 years in persistent–on group, 26 years in hidden–clock group	Laboratory	10, 20, 30 s	2-back	Yes

^a^Longer reaction times in short delay, this paper was not included in the meta-analysis since the full text was written in Chinese

^a^Check the clock more frequently in short time interval

Note: In the column “Effect of time intervals on PM” a “Yes” indicates a decline in performance for the longer delays relative to the shorter delays

*N.R.* = the comparison of PM for the two different time intervals was not reported

^a^ Better PM performance over long duration

^b^Younger adults showed worse performance in long intervals compared to short intervals. Older adults only showed

a decrease in performance for the longest delay of 28 days

^c^Better PM performance over long delays in young adults and higher performance in short delay tasks in older adult

^d^This paper was not included in the meta-analysis due to a lack of statistical information needed to calculate the effect size

**Table 2:** Description of studies investigating different time intervals in time-based prospective memory (PM) tasks in clinical populations

**Study**	Population	Age range (average age in clinical/healthy populations), years	Effect of time intervals on PM	Note
Aronov et al., 2015	Older adults with mild cognitive impairment, subjective cognitive decline, and healthy older adults	80.5	N.R.	Worse performance in long time interval compared to short time interval^1^
Belmar et al., 2020	MCI vs. healthy individuals	(69.2/66.3)	Yes	Worse performance in short time intervals in MCI group compared to healthy older adults
Carlesimo et al., 2004	Head injury patients vs. healthy individuals	(27.4/~27)	Yes	Worse performance in short compared to long time intervals in both groups
Huber, 2022	MCI vs. healthy individuals	(72.26/70.30)	N.R.	Worse performance in long compared to short time interval only in MCI group^1^
Levent & Davelaar, 2022	Drug users vs. non-drug users	18–50+	N.R.	Worse performance in long compared to short time interval in drug users and better performance in long compared to short time interval in non-drug users^1^
Li et al., 2013	Depressed vs. healthy individuals	(17.81/18.16)	N.R.	Worse performance in long time interval in depressed compared to healthy individuals
Miller et al., 2014	Patients with MS vs. healthy individuals	(45.5/38.9)	N.R.	Worse performance in long time interval in patients with MS compared to healthy individuals
Morgan et al., 2012	HIV patients vs. healthy individuals	(44.7/42.7)	N.R.	Worse performance in long time interval in HIV patients compared to healthy adults
Radford et al., 2011	Neurological patients vs. Healthy individuals	(43.6/33.3)	N.R	Worse performance in long compared to short time interval in both groups^1^
Sullivan et al., 2022	Older adults and HIV patients	HIV patients: 18–67 Older adults: 50–91	N.R.	- Worse performance in long compared to short time interval in both groups^1^

*Note:* All studies in Table 2 used the MIST as the Time-Based PM task, with a duration of 2–15 min and a word search as the ongoing task except Carlesimo et al., 2004

N.R. = the comparison of PM for the two different time intervals was not reported

^1^Based on descriptive statistics

### Effects of varying time intervals on time-based PM performance

For our discussion of the effects of varying time intervals on time-based PM performance itself (i.e., did they perform the task?), the findings for healthy individuals and clinical individuals are summarized separately, followed by an analysis of effect sizes for these studies.

**PM performance: Healthy individuals**. Two out of the 15 studies on healthy populations did not reveal a statistically significant effect of time interval on time-based PM performance (Conte & McBride, 2018; Stone et al., 2001). The first study, by Stone et al. (2001), differs from many of the studies discussed below, in that participants had multiple different target time intervals to complete at the same time. In the Stone et al. study, the ongoing task consisted of six trials – each lasted 7 min – in which participants were required to route airplanes through the circuits of waypoints in a simulated air traffic control task. The time-based PM task was embedded in the ongoing task and the participants were instructed to reroute airplane X to # (e.g. “reroute airplane A to 3”) after 1 min, 3 min, or 5 min has elapsed. Each ongoing task trial contained three PM intentions (i.e., all three time intervals) and instructions for each PM intention were presented separately at the start of each trial. The order in which the instructions for each of the three time intervals were presented were random for the participants. As each ongoing task trial was 7 min long, three time intervals were overlapped with each other to fit with the ongoing task duration. In addition, the difficulty of the ongoing task was modulated, and each participant performed the ongoing task with two complexity levels. As a result, the study had two within-subject variables (i.e., ongoing task difficulty and time intervals). Findings of the study demonstrated that time-based PM performance is similar through different time intervals.

In keeping with the studies discussed below, Conte and McBride (2018) used a between-subjects design to measure the effects of three time intervals (i.e., 1, 3, 6 min) and two PM types (i.e., event- vs. time-based PM tasks). Participants were randomly assigned to one of seven groups (i.e., three time intervals and two PM type groups and a control group with no PM intention). The ongoing task was a lexical decision task in which participants were told to determine whether each letter string presented on the screen was a word or non-word. The results again showed no significant effect of time intervals on time-based PM performance.

In contrast to the null effect of varying time intervals in the Stone et al. (2001) and the Conte and McBride (2018) studies just described, 12 other studies have shown a significant effect of different target time intervals on PM performance (one study did not statistically report the effect of different time intervals on PM performance (D’Souza et al., 2024)). A study by Nigro et al. (2002) used a between-subjects design and examined PM performance for children aged 7 to 11 years and were given the time-based PM task to remind the experimenter to make a phone call after 5 (short-delay condition) or 10 min (long time interval). The time-based PM task was embedded in an ongoing task, which was performing mathematical operations and puzzles. The results showed that children had better time-based PM performance in the short time-interval condition compared to the longer interval. More specifically, in the short time interval, 11 of 20 participants performed the PM task at the target time; however, this number decreased to only one participant on the long-delay condition.

Another example comes from the investigation by Jie et al. (2016) of participants’ time-based PM performance in two experiments. In the first experiment, participants were instructed to perform the PM task at 2- or 8- or 15-min target time intervals. Findings are aligned with the previous study supporting worse PM performance in a longer time-interval condition. In the second experiment, the 8-min time-interval condition was deleted. In addition, the duration of the filler task – which is the duration between getting PM instructions and starting the ongoing task – was changed. That is, in the short filler task condition, the PM instruction was presented for the participants and after completing the filler task for 2 min, participants were required to perform the PM task either in a 2- or a 15-min PM target time interval. In the long filler task condition, the duration of the filler task was 15 min, followed by either a 2- or a 15-min PM target time. As a result, the study had a 2 (filler task durations: 2-min vs. 15-min) × 2 (target time intervals: 2-min vs. 15-min) between-subjects design. Results of the second experiment showed that participants performed better in the short PM target time condition when the filler task was long. In the short filler task condition, the PM performance did not differ across short and long target time intervals.

In alignment with these studies, three studies investigated time-based PM performance over shorter time intervals. In a study conducted by Park et al. (1997), participants were asked to perform a time-based task every minute or every 2 min across the 12-min time frame while they were doing a working memory task. The results indicated that both younger and older adults had worse time-based PM performance in the 2-min time-interval condition compared to 1-min condition. Although this study revealed better performance in the short time-interval condition (with 12 PM responses) compared to the long time-interval condition (with six PM responses), it remains unclear whether enhanced performance in the short condition is because of the shorter durations between target times or a practice effect.

In another study with identical time intervals (i.e., 1 and 2 min), Bastin and Meulemans (2002) conducted an investigation into the performance of both younger and older adults in a time-based PM task while they were doing a word recall task. Similar findings emerged, with a trend for better PM performance in shorter time intervals compared to longer durations.

Tsai and Gilbert (2024) investigated the effects of 10-, 20-, and 30-s delays on time-based PM performance. Participants performed a two-back task and were required to press a specific key on a keyboard after 10, 20, and 30 s had passed (a within-subject design). The results indicated that longer time intervals decreased PM performance only for the first PM response. However, for the total PM targets, the delay did not influence PM performance. Alongside the five laboratory studies discussed above (Bastin & Meulemans, 2002; Jie et al., 2016; Nigro et al., 2002; Park et al., 1997; Tsai & Gilbert, 2024), studies with other experimental designs showed similar results.

McBride et al. (2013) used a naturalistic between-subjects design to examine the effects of different time intervals on time-based PM performance across age. Younger and older participants were assigned to one of the five different time intervals (i.e., 1, 2, 5, 14, or 28 days) and were instructed to mail a postcard to the researcher on a specific day. As the study measured a naturalistic PM task, the ongoing tasks were participants’ daily activities. The study showed that younger adults’ performance in the time-based PM task decreased as the number of days until the post card was to be mailed increased; however, older adults’ performance diminished only at the 28-day interval relative to all shorter intervals. In other words, older adults’ performance did not decrease across the first four time intervals (i.e., 1, 2, 5, and 14 days). In summary, the results of the study indicated that younger adults’ performance in time-based PM tasks are negatively affected by increasing the delay intervals, while older adults’ performance was only affected at the longest delay.

In a recent study, Black and McBride (2024) replicated the findings of McBride et al. (2013) by investigating PM performance across time intervals of 1, 3, and 6 days. Participants (i.e., undergraduate students) were instructed to send a text message to the experimenter after a specific delay interval had passed. The results indicated that time-based PM performance decreased as the time interval increased. In contrast to McBride et al.’s results, Schnitzspahn et al. (2020) conducted a study using a naturalistic time-based PM task with time intervals of 1 and 3 days. In their study, participants (i.e., young and older adults) were required to send a message to the experimenter and make a phone call after 1 and 3 days, respectively. Although this study did not report a statistical analysis of possible effects of short and long delays on PM performance, the descriptive statistics showed that, at least numerically, younger adults performed better over longer time intervals compared to shorter ones. However, older adults demonstrated a reverse pattern numerically, with better performance over shorter delays compared to longer durations.

One way to benefit from a naturalistic setting and at the same time reduce its shortcomings (i.e., external aid availability) is using virtual reality. This technique is applied in Kourtesis et al.’s (2021) study by using a within-subjects design in which participants completed time- and event-based PM tasks in short (i.e., 15–30 min) and long (i.e., 45–60 min) time-interval conditions while they were engaging in an ongoing activity (i.e., shopping, cooking, and making breakfast in a virtual environment). In this experiment, PM performance was assessed in two conditions: in the first condition participants received a prompt up to three times, which reminded the individuals of the PM task if the PM intention had been forgotten; in the second condition, participants did not receive reminders. In both the condition with reminders and the condition without reminders, performance was better for PM tasks performed at shorter time intervals relative to PM tasks performed at longer time intervals.

In addition to self-designed tasks, which can vary widely and complicate comparisons between studies, there are standardized instruments for measuring prospective memory. One such instrument, used in both clinical and healthy populations, is the Royal Prince Alfred Prospective Memory Test (RPA-ProMem) (Radford et al., 2011). The RPA-ProMem consists of four parts: the first two parts are short-term, lab-based tasks, with part 1 focusing on time-based PM and part 2 on event-based PM. In these tasks, participants are required to remind the experimenter to do something in the near future (within 15 min) or to respond to an environmental cue for the event-based task. The third and fourth parts are long-duration PM tasks to be completed in a naturalistic setting. The third part involves an event-based task, requiring participants to respond to an environmental cue, while the fourth part is a time-based task, where participants must remember to perform an action at a specific future time (after 1 week).

The RPA-ProMem does not focus on comparing time-based PM tasks over short and long time intervals. As a result, none of the studies using this task statistically compared participants’ time-based PM performance across different durations. Only a few studies (Cantrelle et al., 2020; Green & Loprinzi, 2019) reported time-based PM performance separately for short and long delays. In the first study, Cantrelle et al. (2020) examined whether engaging in open-skilled (Racquetball) versus closed-skilled (treadmill) exercise influences memory function. Participants were required to tell the experimenter what their last meal was 20 min after finishing exercise (either Racquetball or treadmill). Additionally, they were supposed to send a note about the weather to the researcher 24 h after the experiment. Descriptive statistics showed that participants in both conditions (Racquetball and treadmill) performed better, at least numerically, over long intervals compared to short durations.

Similar to Cantrelle et al.’s study, Green and Loprinzi (2019) explored the impact of exercise on memory function in healthy young adults. Participants in the exercise group were asked to walk on a treadmill for 15 min, while those in the control group were instructed to sit for the same duration. For the short time-based task, participants were required to inform the researcher what they had for lunch 20 min after completing their activity. Additionally, they were asked to mail a card describing the weather to the researcher 1 week after the experiment. Contrary to Cantrelle et al.’s (2020) findings, participants in Green and Loprinzi’s study showed a numerical advantage for the short-delay task compared to the long-duration task.

The rest of the studies using RPA-ProMem have not separately reported time-based PM performance over short and long durations. However, we contacted the authors to request the statistical information needed to estimate effect sizes. As a result, we were able to include these studies in our meta-analysis (Aronov et al., 2015; D’Souza et al., 2024; Huber, 2022; Levent & Davelaar, 2022; Radford et al., 2011).

Finally, the youth version, MISTY, of the MIST task has been used to measure time-based PM in healthy children (Mills et al., 2021). The MIST is described in greater detail in the next section as it is more consistently used in studies with clinical populations. Healthy children performed better in short time intervals (i.e., 2 min) compared to a long-delay condition (i.e., 10 min) when MISTY was used to measure the PM performance.

The use of different time intervals and other varying design aspects in the aforementioned experiments prevent us from making a single unqualified statement about the effect of delay on time-based PM performance. However, the design differences point to a potentially important factor in determining when varying the target time interval will affect time-based PM performance; namely, what is a long delay in some studies may match a short delay in others. For example, the long delay condition in the Stone et al. (2001) study (i.e., 5 min) is considered as a short delay in the Nigro et al. (2002) experiment.

#### PM performance: Clinical populations

Beside the studies previously mentioned, there are several studies with clinical populations that use the MIST collection of PM tasks (Woods et al., 2008). The MIST, which was developed and standardized expressly for use in clinical evaluations, includes eight PM tasks (four time-based and four event-based tasks) using two time intervals (i.e., 2 and 15 min) and two response modalities (verbal vs. physical response), all to be completed while participants are engaged in an ongoing word search task. Each time interval comprised four trials that were balanced on verbal versus physical response and event- versus time-based cues. One example is the PM task to remind the researcher to have a short break in 15 min (i.e., time-based, 15 min, verbal task). The MIST also includes a retrospective memory task to assess whether participants remember each of the PM tasks at the end of the experiment. Finally, the MIST includes a delayed naturalistic PM task requiring participants to call the experimenter 24 h after the experiment and report the number of hours they slept the previous night.^2^

There are some studies using MIST to compare the PM performance over short and long delays. For example, Weinborn et al. (2011) compared PM performance (event- and time-based PM) in two time intervals in ecstasy users, high-risk alcohol users, and healthy individuals. Results showed that ecstasy users performed worse than the two other groups in time-based PM task in a 15-min time interval. Additional studies have applied the MIST to measure PM performance in HIV patients (Avci et al., 2016; Morgan et al., 2012; Poquette et al., 2013; Sullivan et al., 2022), depressed patients (Li et al., 2013), individuals with multiple sclerosis (MS) (Miller et al., 2014; Weber et al., 2019), older adults with activities of daily living problems (Tierney et al., 2016), and individuals with Mild Cognitive Impairment (MCI) (Belmar et al., 2020). Overall, studies using the MIST have shown that time-based PM performance decreases in longer delay conditions in clinical populations compared to healthy individuals. One exception is a study conducted by Belmar et al. (2020) in which PM performance in a MCI population and healthy individuals was compared using MIST. Results of the study indicated that both groups had better performance in short-delay intervals (e.g., 2-min tasks) compared to long durations (e.g., 15-min tasks). In terms of group interaction, the MCI group showed lower scores in short interval tasks compared to the control group when contrasted with longer time intervals. However, there was no difference between MCI and healthy individuals’ performance in longer time interval tasks. Generally speaking, studies using the MIST are not reporting separate analyses of the effects of time interval on time-based PM tasks alone. One exception in reporting comes from the Weber et al. (2019) study indicating worse time-based PM performance in a long-delay condition for healthy individuals.

An exception to studies using MIST for measuring PM performance in clinical populations is Carlesimo et al. (2004). In this study, healthy individuals and patients with closed brain injuries were asked to perform specific actions, such as turning off a computer after a designated time had elapsed, following short (10 min) or longer (45 min) delay intervals. To engage participants’ attention, computer-based vigilance tasks were used as ongoing activities. Consistent with the findings of Belmar et al. (2020), PM performance was better after longer delays compared to shorter ones in both groups. In summary, while studies involving clinical populations have not directly investigated the effects of delay on time-based PM, it is the case that differences between the control and clinical groups are found to be larger at the longer delays, suggesting a role for the longer time interval in explaining performance in these tasks.

#### PM performance: Evaluation of effect size

To include as many papers as possible in the meta-analysis, we reached out to the authors to ask them about their statistical information to be able to conduct an evaluation of effect sizes. Finally, we had enough statistical information (i.e., sample size, mean, and standard deviation of time-based PM performance in both short and long time intervals) from 22 studies with 93 effect sizes.

We assessed the methodological quality of the papers using the Downs and Black checklist. This checklist consists of 27 items that evaluate methodological quality across four domains: reporting, external validity, internal validity, and power. Scores range from 0 to 28, with one point assigned to each item, except for item 5, which can receive up to 2 points. Based on this checklist, the studies demonstrated good quality, with a mean score of 19.68 (SD = 1.32). All studies were within three standard deviations of the mean, so we did not exclude any of them from the meta-analysis.

**Fig. 2:**
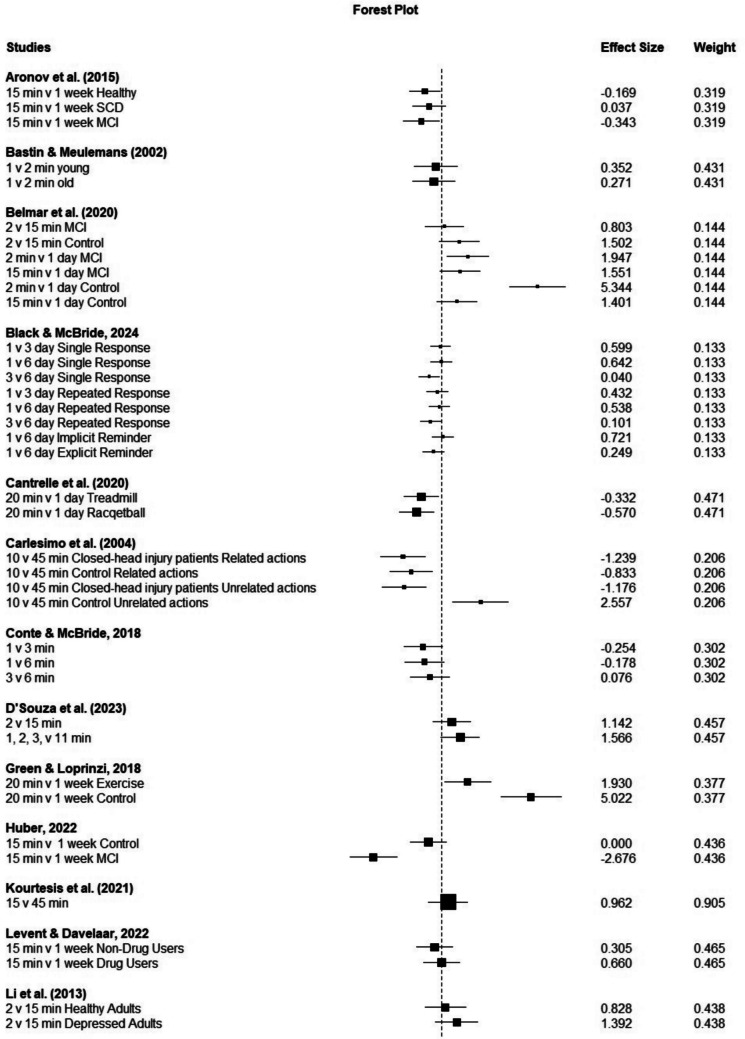

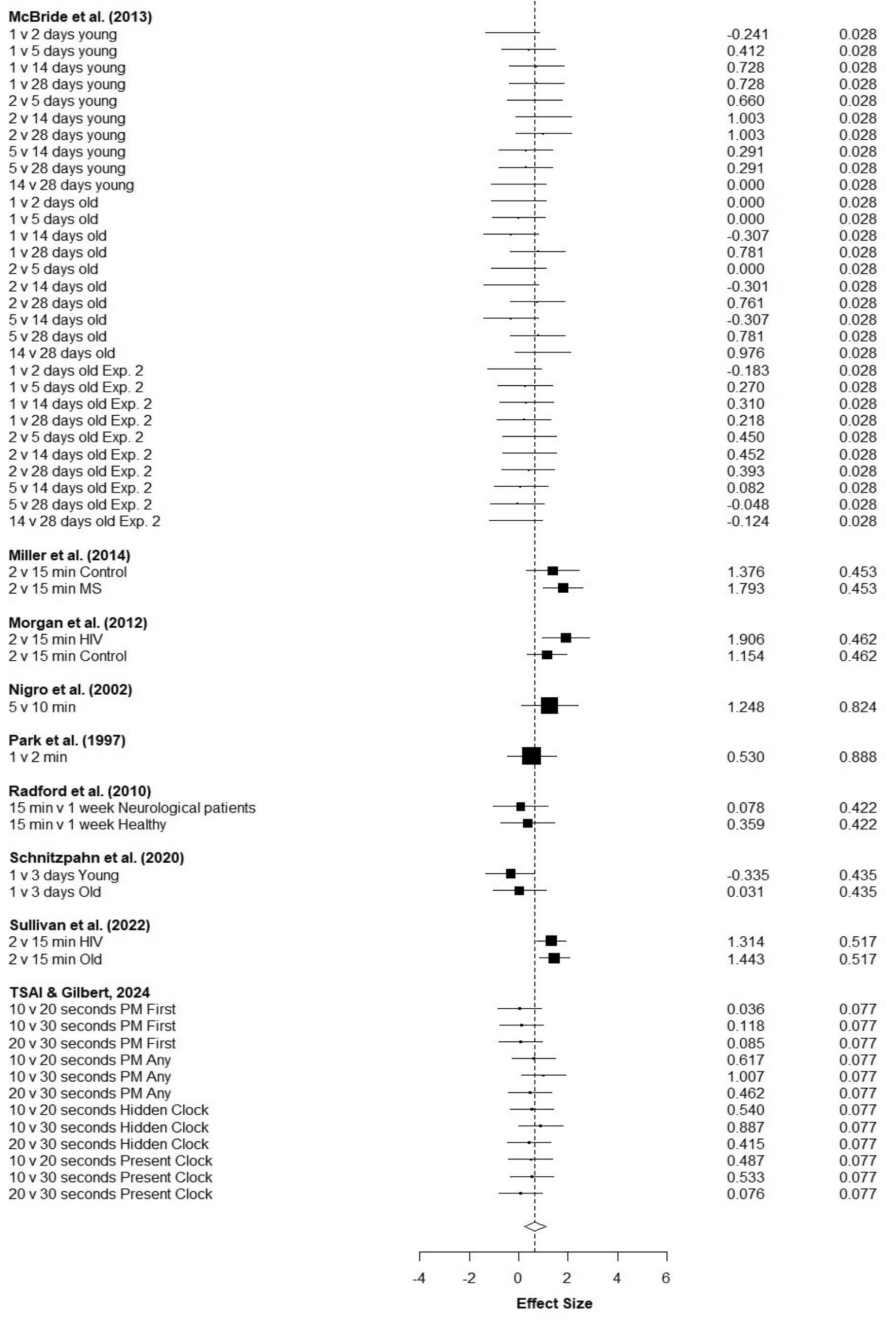
Forest plot for observed effect size of time-based prospective memory performance in short and long time intervals. The effect size (shown as squares) and confidence intervals (represented by horizontal lines) for each individual study, along with the pooled effect size (a diamond at the bottom of the plot) is presented. Points to the right of zero are in favor of short delays

To investigate the effects of different time intervals on time-based PM performance, we conducted a meta-analysis using RStudio software (version 2023.06.0–421.0). The analysis utilized the robumeta, metafor, and dplyr packages. An overall calculation of effect size for time-based PM performance (using the random-effects model) across all included studies (studies with healthy and clinical populations; both laboratory and naturalistic studies) indicated that time interval had a significant effect (Hedge’s g =.67, 95% CI:.24, 1.09, *p* <.001, ${I}^{2}$ = 80.46) on time-based PM performance (see Fig. 2,^3^), with better performance in shorter time intervals. Results of studies with only healthy individuals with 79 effect sizes have shown an effect size of.75 (95% CI:.36, 1.13, *p* <.001, ${I}^{2}$ = 76.95), also in favor of shorter time intervals.

To assess the robustness of the meta-analysis results, a sensitivity analysis was conducted by varying the assumed value of the within-study correlation (*ρ*) from 0 to 1. The estimated intercept coefficient remained consistent across all values of *ρ* (*β* = 0.667, *SE* = 0.204). The between-study variance (*τ*^*2*^) showed minimal variation, ranging from 0.870 (*ρ* = 0) to 0.875 (*ρ* = 1), indicating that the overall findings were stable across different assumptions of within-study dependence.

Since existing studies differ in terms of their design (i.e., lab-based vs. naturalistic-based time-based PM tasks), we investigated the possible moderation role of design in the effects of different time intervals on time-based PM performance. Results indicated that there was no evidence of a significant moderation effect for either lab-based (*p* =.841) or naturalistic-based (*p* =.368) PM tasks.

**Fig. 3:**
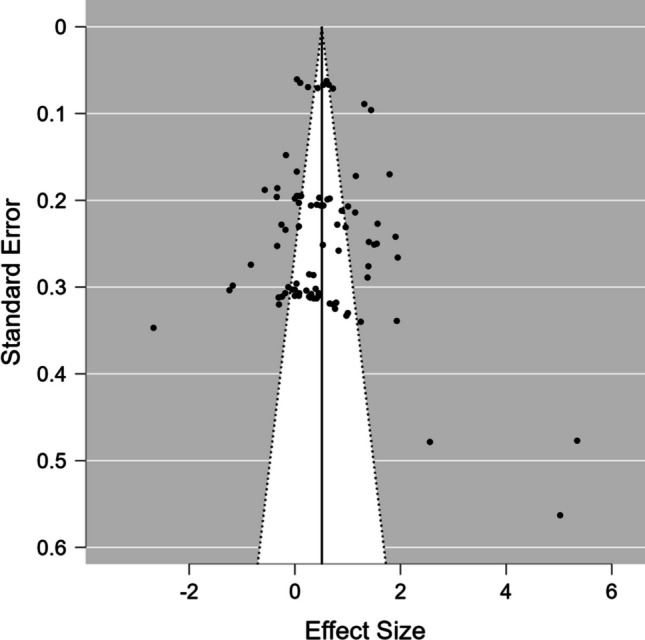
Funnel plot for available study results. The effect sizes of individual studies and standard error are on the horizontal and vertical axes, respectively

In addition, the result of heterogeneity was significant (intercept = 2.605, *p* =.009) indicating that there is a risk of publication bias (Fig. 3,^4^). To explore the impact of potential missing studies, we did a trim-and-fill method. However, this method did not change the overall effect size meaning the observed publication bias does not change the overall effect size.

Furthermore, a Fail-safe N analysis was conducted to evaluate the robustness of the meta-analytic effect size. The results indicated that 19,908 null studies would be required to raise the observed significance level (*p <*.001) to exceed the threshold for statistical significance (*p >*.05). This large Fail-safe N suggests that the meta-analytic findings are robust and unlikely to be influenced by missing studies alone.

Since the RPA-ProMem instrument measures short and long time-based tasks in laboratory and naturalistic settings, respectively, we were concerned that including these studies in the meta-analysis might introduce additional variability. This variability could arise because it would be difficult to separate the effect of delay from the different settings in which the PM tasks were performed. To ensure that including these papers did not influence our results, we conducted the meta-analysis excluding studies that used this instrument. The results, after excluding those effect sizes, remained significant, showing that PM performance was better under short time intervals compared to longer delays (Hedge’s g =.64, 95% CI:.24, 1.03, *p* <.001, ${I}^{2}$= 73.15).

#### Effects of varying time intervals on clock-checking behavior

Among studies that examined the effects of different time intervals on time-based PM, only three studies investigated clock-checking behavior, with two of these involving healthy individuals. In Conte and McBride (2018), participants were instructed to say to the experimenter “time check” when they wanted to check the clock, and the elapsed time was told to them. Results indicated that participants checked the time more frequently as they neared the target time in each time-duration interval (i.e., 1, 3, 6 min). However, the effect of different time intervals on the pattern and frequency of clock checking was not significant. On the other hand, Nigro et al. (2002), in a between-subjects study, investigated the clock checking pattern in 5- and 10-min time intervals and showed a contradictory result. That is, short and longer time intervals showed different clock-checking patterns; in the short time-interval condition, participants checked the clock more frequently than did participants in the longer time-interval condition.

In one clinical study reporting clock checking, clock checking was investigated across HIV patients and healthy individuals (Doyle et al., 2013) using MIST to measure prospective memory performance in 2- and 15-min time intervals. Results revealed that HIV patients checked the clock less frequently than healthy individuals, and the frequency of clock checking was positively related to the overall time-based PM performance. In addition, there was a positive correlation between clock checking and performance in the 15-min time interval.

Although it is difficult to make a conclusion about the effects of short versus long time intervals based on findings of only three studies, it seems that clock-checking behavior could be affected by different delay intervals when the duration of the longer time interval is at least 10 min. In other words, as it is demanding to monitor the clock in durations over 10 min, participants do not check the clock frequently when they are performing a time-based PM task with a long delay.

#### Effects of varying time intervals on ongoing task performance

To examine the cost of the PM task in different time intervals, Jie et al. (2016) investigated reaction times of ongoing task trials. As reported in their abstract, results indicated that in a short-delay condition, participants had longer ongoing task reaction times. In another study, Conte and McBride (2018) examined 25 trials before the target time to investigate the effects of different time intervals on the ongoing task in healthy individuals across three conditions (i.e., event-based, time-based, and control). Results indicated a longer ongoing task reaction time in a time-based PM condition compared to the control condition only in longer time intervals. In contrast, Conte and McBride (2018) also investigated the effects of the PM tasks on the ongoing task accuracy and results demonstrated no significant effect and performance in the ongoing task was similar in conditions with and without performing the PM task.

Based on the abovementioned findings and results related to the PM performance and clock-checking pattern, we can conclude that as participants check the clock more frequently in short time intervals, they have better performance in the PM task. However, the cost of frequent clock checking is longer reaction times on the ongoing task. Thus, it seems that participants sacrifice their ongoing task performance in order to complete the PM task when they are performing a time-based task with a short time interval.

### Discussion

As summarized in the first section of this review, longer time intervals are associated with poorer PM performance. Most previous studies attributed the worse time-based PM performance in longer time-interval tasks to deficiencies in monitoring behavior, arguing that over a longer period of time, consistent time monitoring is more difficult due to the cognitive demands of monitoring while performing other activities. This has been proposed as an explanation for why time-based PM tasks in longer time intervals are more susceptible to the effects of drug use (Weinborn et al., 2011), HIV (Morgan et al., 2012), and depression (Li et al., 2013), suggesting that clinical populations are less likely to use their attentional resources to monitor a task for longer periods of time. As discussed above in the summary of empirical findings, previous studies produced varying results regarding clock checking during short and longer time intervals; while Conte and McBride (2018) indicated that the frequency of clock checking is not affected by different time intervals, Nigro et al. (2002) showed that clock-checking frequency decreases as time interval increases. Although Nigro et al. attributed their findings to participants’ engagement in the ongoing activity, time perception, or internal time processing, might play a role.

Although the Test-Wait-Test-Exit (TWTE) model can explain the clock checking pattern during a time-based PM task, it does not clarify what happens during the “wait” phase and how individuals come to a decision that the appropriate time is reached or decide that the clock should be checked again. Another model that can explain the internal time processes during the “wait” phase is the attentional-gate model (Zakay & Block, 1996).

#### Attentional-gate model

The attentional-gate model (Zakay & Block, 1996) examines the underlying mechanism of the internal clock in time-related tasks and proposes a critical role of attention in such decisions. This model considers four elements for internal time processing: pacemaker, attentional gate, switch, and cognitive counter. Pacemaker is the first element producing a series of pulses which are affected by an organism’s arousal level. The gate opens when an organism pays attention to the timing task. The more attention that has been directed to the temporal task, the wider the gate will become, and the more pulses will be transmitted from the pacemaker to the cognitive counter. The role of “switch” in this model is opening and closing the pathway (the pathway between the attentional gate and the cognitive counter) when a stimulus alarms the initiation or termination of a temporal judgment. Each time the switch opens, the cognitive counter is set to zero to be ready for a new temporal decision. It seems that the TWTE and attentional-gate models complement each other; while the first model explains external time monitoring, the second one addresses internal time processing.

The TWTE and attentional-gate models, taken together with the results of the literature review just presented, suggest an important role for internal time processing – or time perception – in understanding time-based PM performance. This leads to the second review.

## Review 2: The role of time-perception in time-based PM

To our knowledge, only one study directly investigated external versus internal time processing in time-based PM (Huang et al., 2014). In this study, participants completed a lexical decision task as an ongoing task in the first block. In the second block, a PM task was added to the ongoing task in which participants were required to press a key on a keyboard after 11 min from the beginning of the second block. In the first experiment, participants had no restriction for clock checks; however, in the second experiment, they were discouraged from checking the external clock for performing the PM task. Results indicated that internal time monitoring compared to external time monitoring (clock checks) negatively influences the ongoing task performance. This finding shows the occupation of attentional capacity during internal time monitoring. This result is aligned with studies indicating decreased PM performance in clinical populations only in longer time intervals. In other words, when clinical disorders result in less available cognitive capacity, participants are less able to benefit from internal time monitoring, negatively affecting performance in high demanding cognitive tasks (e.g., a longer time-based PM task).

While the Huang et al. (2014) study is the only published study of which we are aware that compared time-based PM when participants were able to freely monitor an external clock versus when participants were discouraged from checking the external clock, another approach to understanding the role of internal time processing is to consider studies that have directly evaluated time-perception and time-based PM in the same study. These types of studies are the focus of our second review.

### Methods

As in the first review, two authors (FFM and PDL) independently conducted literature searches and compared results to determine the number of eligible studies. Any discrepancies were resolved through consensus. We reviewed published studies in three electronic databases: PsycINFO, PubMed, and Scopus from inception to December 2024. The following keywords were used to find relevant articles: “time-based prospective memory” OR “time based prospective memory” OR “time-based PM” OR “time based PM” AND “time perception” OR “time estimation” OR “time production” OR “time reproduction” OR “prospective timing” OR “retrospective timing” OR “time discrimination.” All studies were imported into EndNote (version 21) for duplicate checking and further examination.

### Results

**Fig. 4:**
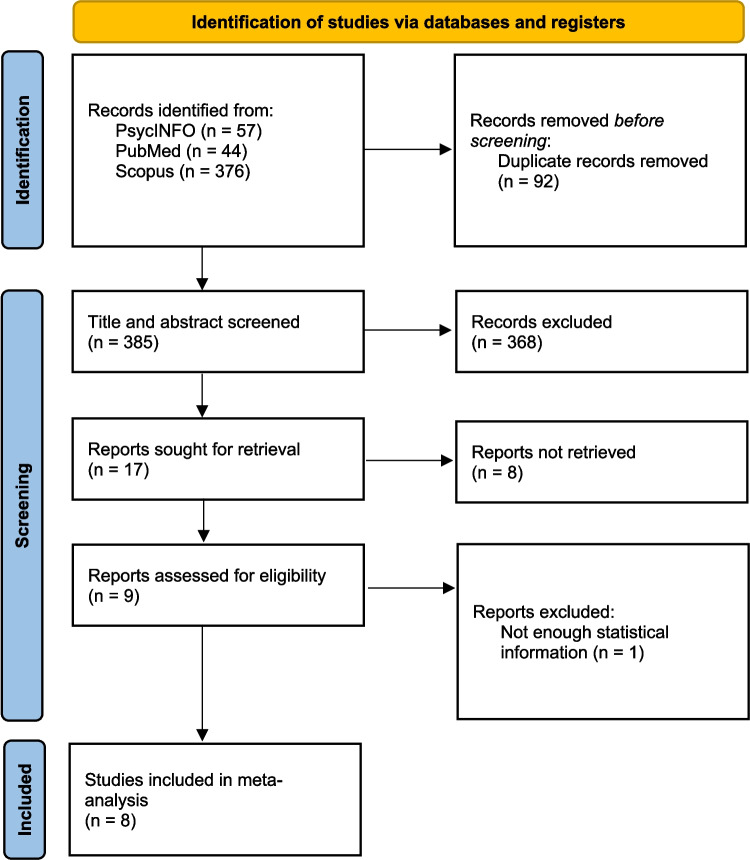
Flow chart showing article retrieval

Figure 4 represents the flow chart of the article retrieval process. A total of 477 relevant papers were found, including 57 from PsycINFO and 44 from PubMed, and 376 from Scopus. After removing repeated studies, the number of papers reduced to 385 studies. Considering their titles and abstracts, 17 papers were selected. After reading the full text of papers, nine papers were selected, and eight studies were selected for the final meta-analysis.

Table 3 presents the studies that have examined the relationship between time perception and time-based PM performance in healthy and clinical populations. As with the first review, we summarize the overall findings, beginning with consideration of studies focused on healthy individuals, followed by discussion of studies including clinical populations. The results of meta-analysis are then provided.

**Table 3:** Description of studies investigating the relationship between time-based prospective memory (PM) performance and time perception

Study	Population	Time-based PM intervals	Type of time perception task	Time perception durations	Ongoing task	Relationship between time-based PM and time perception performances	Relationship between clock-checking and time-perception performances
Doyle et al., 2015	HIV+ with HAND, HIV+ without HAND, healthy individuals	2- and 15-min intervals	Time production task	15, 30, 45, 90 s	Word search	Yes	N.R.
Labelle et al., 2009^**2**^	Healthy individuals	30, 60, 90 s	Time production	30, 60, 90 s	Category member decision task	No	Yes
Mackinlay et al., 2009	Healthy children	Every 2 min	Verbal/retrospective estimation task	2 min	1-back picture task	Yes	No
McFarland and Glisky, 2009	Healthy individuals	Every 5 min	Verbal estimation/time production tasks	27 s/10 s	Computerized general knowledge questions	No	N.R.3
Mioni and Stablum, 2014	Healthy individuals	Every 5 min	Time reproduction task	4, 9, 14 s	Watching a movie	No	Yes
Mioni et al., 2012	Adults with and without TBI	Every 5 min	Time reproduction task	4, 9, 14 s	Watching a movie	No	Yes: Only in healthy individuals
Mioni et al., 2017	Children with and without ADHD	Every 2 min	Time reproduction task	4, 9, 14 s	Watching a movie	Yes: Only in healthy children	N.R.
Mioni et al., 2019	Healthy individuals	Virtual week	Time reproduction task	15, 30 s	Computerized board game	No	Yes
Vanneste et al., 2015	Healthy younger and older adults	Every 1 min	Time production task	5, 8, 18, 23 s	Word Categorization	Yes	Yes

^1^Gan and Gue (2019), Waldum and McDaniel (2016), and Woods et al. (2009) were not included in the table since the first two studies did not report the correlation between time-based PM and time perception statistically, and the last one reported the relationship between time estimation and loss of time error.

^2^ Not included in meta-analysis since the relationship between time-based PM and time perception has not been reported

^3^ Not reported

As a preview, the findings are at this time mixed, without a clear answer regarding the relationship between time-perception and time-based PM, but – as will be addressed in the discussion section for this review – this summary points to possible explanations for these mixed findings. Because the conclusions from this review rely on understanding methodological differences between different studies, we include fairly detailed consideration of the methods used in various studies.

#### Relationship between time-based PM and time perception in healthy individuals

As far as we know, half of the studies investigating the relationship between time-based PM and time perception found no relationship between these two cognitive functions (Labelle et al., 2009; McFarland & Glisky, 2009; Morgan et al., 2012; Mioni et al., 2012, 2017, 2019). However, the other studies reported a correlation between these two timing abilities (Doyle et al., 2015; Gan & Guo, 2019; Mackinlay et al., 2009; Mioni et al., 2017; Vanneste et al., 2015; Waldum & McDaniel, 2016).

While all these studies require participants to perform both time-based and time-perception tasks, they utilize different tasks with varying durations, making comparisons between studies difficult. Not only do task durations vary across studies, but the durations of time-based and time-perception tasks also differ within each study. Two exceptions are the studies by Labelle et al. (2009) and Mackinlay et al. (2009), where both time-based and time-perception tasks were set at 30, 60, and 90 s and 2 min, respectively. Although these studies share similarities in using the same durations for both tasks, they showed contradictory results: Labelle et al. found no significant relationship between time-based and time-perception performance, whereas Mackinlay et al. found a significant relationship. Vanneste et al.’s study (2015) also found a significant relationship between time-based PM and time-perception tasks, even though the durations of these tasks were not identical (1 min for the time-based PM task and 18–23 s for the time-perception task).

In other studies, the duration of time-perception tasks was much shorter than that of the time-based tasks. For instance, in the studies by McFarland and Glisky (2009) and Mioni and Stablum (2014), the time-based tasks had a duration of 5 min, whereas the time-perception tasks were 10 and 27 s in McFarland and Glisky’s study and 4, 9, and 14 s in Mioni and Stablum’s study. Both studies found no significant relationship between time-perception and time-based PM performance, although Mioni and Stablum’s study did show a significant relationship between time perception and clock checking. Additionally, Mioni et al. (2019) found no relationship between these two timing abilities even when a more naturalistic time-based task (i.e., virtual week) was used to measure time-based PM performance, and the time-perception task had durations of 15 and 30 s.^5^

##### Summary

In general, there is conflicting evidence regarding the relationship between time-based PM and time perception. These inconsistencies in findings may arise from variations in the durations employed in both time perception and time-based PM tasks, as well as other differences in the methodology utilized.

#### Relationship between time-based PM and time perception in clinical populations

The relationship between PM and time perception has also been investigated in clinical populations. However, as with studies on healthy populations, the findings across different clinical studies are not clear and consistent. For example, some studies found no relationship between time-based PM and time perception abilities, such as Mioni and Stablum (2014), which used a 5-min time-based task and time-perception tasks of 4, 9, and 14 s with individuals with severe traumatic brain injury (TBI); Mioni et al. (2017), which used a 2-min time-based task and time-perception tasks of 4, 9, and 14 s in children with ADHD; and Morgan et al. (2012) and Woods et al. (2009), which used time-based tasks of 2 and 15 min and time-perception tasks of 15, 30, 45, and 90 s in individuals with HIV. In contrast, a significant relationship was found in other studies, such as Doyle et al. (2015), which used time-based and time-perception tasks of 15, 30, 45, and 90 s in individuals with HIV.

Although some studies found no significant relationship between time-based PM and time perception, a significant relationship between time perception and clock checking has been reported. For example, Mioni et al. (2012) demonstrated that healthy individuals checked the clock more strategically than those with severe TBI, with a correlation between time perception and clock checking observed only in healthy individuals. Similarly, Mioni et al. (2017) found that healthy children checked the clock more strategically compared to children with ADHD.

##### Summary

Overall, previous studies indicated that healthy individuals check the clock strategically, fewer in the first part of the PM task and more frequently near the target time, whereas clinical populations do not benefit from strategic time monitoring. In addition, in most studies, clock checking as a method to help participants to successfully perform the PM task is related to time perception; however, the connection between time-based PM and time perception has yielded inconsistent findings.

#### Relationship between time-based PM and time-perception performance: Evaluation of effect sizes

To investigate the overall relationship between time-based PM and time-perception performance, we conducted a meta-regression using JASP software (version 0.17.3.0). To address variations across the reviewed studies, such as differences in the duration of time-based PM and time-perception tasks, participants’ age, methods for calculating time-perception performance, and whether the participants were drawn from a clinical sample or healthy individuals, we excluded the clinical population from further analysis. Our focus was on studies involving healthy populations, and we included eight studies (with 18 effect sizes) that reported either the correlation between time-based PM and time perception (i.e., correlation coefficient) or sample size, mean, and standard deviation of time-based PM and time-perception performance for the meta-analysis. We used Comprehensive Meta-Analysis (CMA) software (version 4) to calculate the effect size and standard error of each study. Since some studies have reported each group’s mean and standard deviation and others have reported correlation coefficients, the calculated effect sizes were not based on the same metrics. To overcome this issue, we used the esc_rpb function in RStudio software (version 2023.06.0 Build 421) to convert effect sizes that were based on the correlation coefficient to Hedge’s g. This function is based on the following formula for the equally sized group (*n*_*1*_ = *n*_*2*_) (Lipsey & Wilson, 2001):

$$\mathrm{Hedges}^{\prime} g=\frac{2r}{\sqrt{1-{r}^{2}}}$$

A different formula should be used for unequally sized groups (Aaron et al., 1998):

$$\mathrm{Hedges}^{\prime} g=\frac{r}{\sqrt{(1-{r}^{2})(\frac{{n}_{1}}{N} \times \left(1-\frac{{n}_{1}}{N}\right))}}$$

To analyze all effect sizes, we used the software JASP. Inference of a random-effects meta-analysis was computed with the restricted maximum likelihood methods. A test of residual heterogeneity yielded a significant result (*Q* = 102.92, df = 12, *p* = <.001). Additionally, we considered two moderators in our analysis based on the different methods used to calculate time-perception performance (e.g., ratio, absolute difference, and subjective time perception) and the participants’ age.

The results indicated a significant, negative correlation between time-based PM and time perception performance in studies that used the absolute difference formula to calculate time-perception performance (i.e., subjective duration – objective duration) [Wald test: *b* = −1.55, 95% CI = −3.23; -.55) z = −2.76, *p* =.006]. However, the relationship was not significant in studies that used the ratio formula (i.e., subjective duration/objective duration) [Wald test: *b* =.16, 95% CI = −1.51; 1.14) z = -.27, *p* =.74] or subjective duration (i.e., only reporting the subjective duration) [Wald test: *b* = -.04, 95% CI = −2.17; 1.42) z = -.41, *p* =.681] to calculate time-perception performance. Regarding the age of the participants, the results showed a non-significant correlation between time-based PM and time-perception performance for both young [Wald test: *b* =.2, 95% CI = −1.78; 1.50) z = -.17, *p* = 0.864] and older adults [Wald test: *b* =.33, 95% CI = −1.98; 1.95) z = -.02, *p* =.988].

**Fig. 5:**
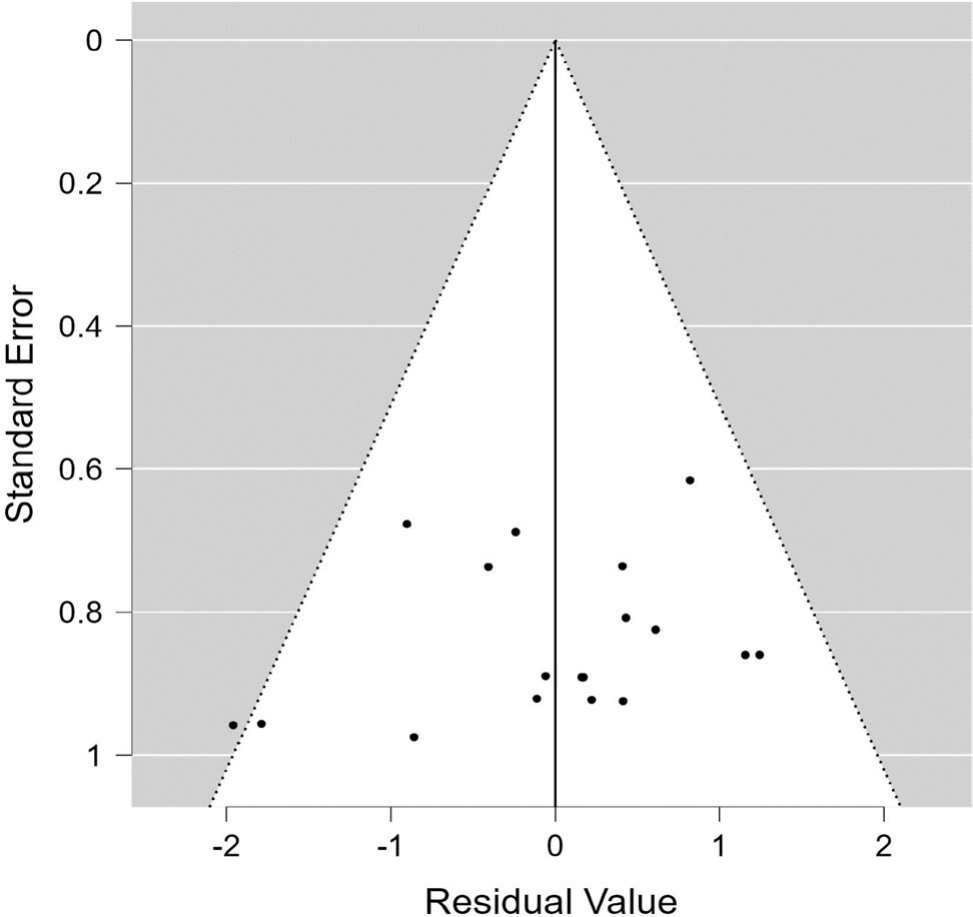
Funnel plot for available study results

The heterogeneity variance was estimated at *τ*^*2*^ = 0.95 (95% CI =.44, 2.89), with an *I*^*2*^ value of 92% (CI = 84–97%), indicating that statistical heterogeneity between studies was significantly large. Although the funnel plot (Fig. 5) and the Kendall test’s result represented no bias in the publication record (*p* =.881), the Egger’s regression test indicated a bias (*p* <.001). To address the publication bias, we used PET-PEESE method. The result of PET model was not statistically significant (*ρ=.*193, 95% CI = -.763, 1.149) suggesting there is no evidence of publication bias.

### Discussion

As noted above, the results of our review of studies investigating the relationship between time-perception and time-based PM are mixed. However, closer examination of the pattern of findings, combined with consideration of different methodological details, raises several points in previous studies worth mentioning. First, previous studies only focused on short durations in the time-estimation tasks although time estimation of short durations only requires short-term memory, whereas durations over 120 s rely on long-term memory (Block & Zakay, 2006). As a result, one limitation of previous studies is the exclusive focus on short durations in time-estimation tasks. It is difficult, perhaps not possible, to investigate the relationship between time-estimation and time-based PM when the duration in the longest time-estimation task was much shorter than the duration of the longer delay PM task. On the other hand, as described above in the results of time-perception studies, when the duration of time-perception and time-based PM tasks were same, there was a positive correlation between these two time-related abilities (e.g., Mackinlay et al., 2009). Hence, one reason for the null relationship between time-based PM and time perception in many previous studies might be due to the methodological approaches adopted to measure timing abilities.

As a counter to this concern, it is important to consider that one assumption in completing a time-based PM task is that when the delay interval is longer than a minute, participants may not estimate the passing time for a long period, but rather unconsciously divide the 5-min interval into to shorter durations^6^ (e.g., 30 s) and check the external clock periodically (Block & Zakay, 2006). If this is the case, previous studies intended to examine the relationship between time-perception and time-based PM performance are perhaps appropriately designed. Thus, it seems that the first step in understanding the role of time perception in time-based PM tasks is understanding individuals’ strategies to perform a PM task in order to appropriately design an experiment to investigate the role of time-perception in time-based PM.

Secondly, some studies revealed a correlation between time perception and clock-checking behavior, showing that monitoring the clock less frequently and more strategically is related to more accurate time estimates (Labelle et al., 2009; Mioni et al., 2012; Mioni & Stablum 2014; Mioni et al., 2019). This finding could be interpreted as an indirect role of time perception in time-based PM. In other words, although in some studies time perception is not a predictor of time-based PM accuracy, it might play an indirect role through clock-checking behavior, since previous studies indicated that strategic clock-checking behavior can predict a successful time-based PM performance (Ceci & Bronfenbrenner, 1985; Zinke et al., 2010).

## General discussion

The primary aim of the current research was to compare and summarize previous studies that examined the impact of different time intervals on time-based PM performance. After reviewing 93 studies and conducting a meta-analysis, it was found that time-based PM performance declines as the duration of the time interval increases. This negative effect of longer time intervals is more pronounced in clinical populations with reduced cognitive capacity to maintain PM intentions over extended periods. There are several possible reasons for the deterioration of PM performance over longer intervals. For instance, participants may forget the retrospective aspect of the time-based PM task (i.e., what needs to be done?) and may also be more likely to forget the PM intention altogether due to the ongoing task occupying their attention.

At first glance, one might be tempted to suggest that the findings of our study appear to be inconsistent with those of Laera et al. (2023), who conducted a meta-analysis of age-related differences in time-based prospective memory performance. Laera et al. found that age-related differences (with poor performance for older relative to younger adults) were greater for time-based tasks at shorter intervals relative to the size of age-related differences at longer intervals. If one assumes that the decrease in the age-related difference at longer intervals is due to improved performance for older adults at longer intervals, then this would seem to contradict our findings of declining performance in longer intervals relative to shorter intervals. However, the results of the Laera et al. study do not speak to the question of whether older adults’ performance increased or decreased from short to long time intervals as their study was not designed to investigate the effects of duration on time-based tasks. A key difference between our study and that of Laera et al. (2023) lies in the designs used in each of the meta-analyses. While we examined whether time-based PM performance differs across short and long time intervals, Laera et al. (2023) investigated how the duration of the PM target time (or time interval) moderates PM performance in older and younger adults. Laera et al.’s finding that the age-related difference in time-based prospective memory performance was reduced for longer durations could be explained in a number of ways (e.g., younger adults show a larger decline from shorter to longer), but this finding does not speak to the pattern of change from short to long intervals.

In addition to differences in design noted above, while Laera et al. (2023) assessed time-based PM performance in studies conducted in laboratory settings, we also included studies that utilized naturalistic PM tasks to explore whether results differ across different task designs. Finally, whereas Laera et al. (2023) primarily focused on age-related differences and the effect of time intervals, our study also examined the role of time perception in time-based PM, an aspect not thoroughly addressed in previous reviews. This provides a novel perspective on current literature. Thus, the findings from these two reviews are not contradictory, but both provide unique and potentially complementary information.

One possible factor contributing to the decline in PM performance during longer time intervals is time perception. We assume that compared to shorter durations, participants tend to rely more on internal time processing over extended periods. However, internal time processing is less accurate than external time processing (i.e., clock checking), making time-based PM performance more vulnerable to impairment in long time intervals.

The second aim of the study was to clarify the relationship between time-based PM and time perception, which has yielded mixed results in the literature. The discrepancies in previous research may be attributed to differences in experimental designs, the duration of timing tasks, and variations in calculating participants’ performance in time perception. The use of different formulas to calculate time perception can lead to contrasting outcomes, making it difficult to determine whether null results in certain studies indicate an actual lack of correlation between the variables or are influenced by the specific mathematical approach used.

Our meta-analysis showed that when time perception was calculated using the absolute difference method, a negative correlation between time-based PM and time perception was observed. However, in young adults, a positive correlation between time-based PM and time-perception performance was found. It is important to note that different calculation methods for time perception can even impact the direction of results, raising uncertainty about whether the negative correlation in our findings truly signifies a negative relationship or is influenced by the chosen mathematical formulas.

### Limitations of existing work

As described in the discussion for the second review, studies of time perception have generally employed a much shorter time interval compared to time-based PM studies. It remains an open question whether this is a fundamental flaw in the design, or a perfectly appropriate approach. The answer to these methodological issues calls for a better understanding of how we might rely on the combined processes of internal time monitoring and external clock checking. This limitation in our basic understanding of the interplay of time perception and clock checking points to future directions for research, as discussed below.

The issue of different time intervals in time-perception studies and time-based PM studies is closely related to limitations in the work reported in the first review covering time intervals and time-based PM. Different studies use widely different time intervals. In other words, there is not a common language of short and longer time intervals in previous studies of time-based PM. Should a 5-min delay be considered as a longer time interval (as in Stone et al., 2001) or a short duration (as in Nigro et al., 2002)? Lack of consensus in scientific terms makes it difficult to arrive at a clear conclusion about the effects of time interval on time-based PM performance.

Another limitation related to time perception involves the absence of uniform approaches for assessing time-perception performance. Determining which method is more reliable or truly reflects time-perception performance becomes challenging due to diverse strategies used to calculate the variable. These variations often result in disparate or conflicting findings, making it arduous to reach a definitive conclusion regarding the relationship between time-based PM and time perception.

The last limitation pertains to the inclusion of studies in both sections of the meta-analysis. As previously noted, the majority of studies on time-based prospective memory have focused on fixed time intervals. Consequently, our access to a substantial pool of papers for inclusion in the meta-analysis was limited. Thus, of the eligible studies, 36 studies were excluded due to insufficient statistical information. This same constraint applies to the second meta-analysis, which relied on only eight studies.

### Directions for future studies

Based on our review, we present suggestions for future studies. First, to examine the effect of time intervals, we need an experiment that covers a greater range of intervals that have been studied in previous laboratory-based experiments from the shortest (i.e., 1 min) to the longest (i.e., 60 min) duration. By doing so, we would potentially be able to determine a distinction between short and longer time intervals in laboratory experiments. Even longer delays are possible using tasks outside of the laboratory, but as a start, examining the effects of delay across a broader range of time intervals in a single study would help to address the apparently disparate findings regarding the effects of interval on time-based PM within laboratory studies.

A second limitation of previous studies is that time intervals in naturalistic tasks (at least a day) are much longer than those in laboratory tasks (limited to a maximum of 45 min). This difference hinders us from determining whether the variations in results are attributed to the task’s environment or its duration. To overcome this issue, we can design an experiment to investigate time-based PM in naturalistic tasks with shorter delays. For instance, instructors could request students to send a summary of the session 1 h after the class has ended.

A third issue that requires consideration is whether there must be a specific minimum interval between short and long delays to highlight the difference. The difference between the short and long delays in previous studies varied from 2 to 15 min in laboratory PM experiments. Is there a minimum difference required to detect a difference in performance? For example, with variations in time intervals of 15 s be too brief to detect a difference? Future research could investigate the overall forgetting curve of time-based prospective memory, including minimum time differences required to detect performance differences.

While most experiments involving PM tasks typically involve a distracting task before the start of the ongoing task in which the PM task is embedded, McBride et al. (2011) conducted a study to investigate the impact of short versus long time intervals in cases where no distracting task intervened between the PM instruction and the start of the ongoing task in an event-based PM paradigm. When using an event-based PM task, as in the McBride et al. study, the delay does not define the PM task in the way that it does in time-based PM. Instead, participants in this study were to make the PM response when the target event, or target images, appeared. The delay in this case is the number of trials or amount of time of the ongoing task preceding the first appearance of a PM target event. The findings of McBride et al.’s study revealed that, even in the absence of a distracting task, performance was better during shorter delay intervals compared to longer durations.

This points to a fourth area for future investigations: determining possible effects of the distractor delay between the time the PM instructions are given and the time that the ongoing task begins on whether effects of the time interval will emerge. Will this additional source of delay impact the effects of varying the time interval of a time-based PM task? Previous studies indicated that even the delay from when the PM instruction is given to participants to the moment the ongoing task with the embedded PM task begins (the delay in which distractor tasks are presented) influences their performance such that when the distracting task was long (e.g., 15 min) time-based PM performance was better under the short time-interval condition rather than a longer time interval (Jie et al., 2016). However, when the distracting task was short (e.g., 2 min) there was no difference in time-based PM performance between short and long time-interval conditions (Jie et al., 2016). Additional research is needed in this area as well.

The fifth suggestion pertains to clock-checking behavior and its relationship to time-based PM performance and time perception. Given that several previous studies reported an association between time perception and clock checking, and a non-significant correlation between time perception and time-based PM performance in the same experiment (see Labelle et al., 2009; Mioni & Stablum, 2014; Mioni et al., 2019), it is possible that clock checking as a variable related to both time-based PM and time perception could act as a mediator.

The next recommendation deals with discrepancies in measuring time-perception performance. Earlier studies typically employed one or, at most, two methods to measure participants’ time perception. To address this, one potential recommendation is to calculate time-perception performance using all available approaches and then correlate each method with time-based PM to examine the outcomes. If consistent results emerge across multiple studies, we can make a conclusion about which approach is more reliable than others.

Finally, based on findings of previous studies, we assume that the role of time perception in time-based PM tasks depends on the duration of the time interval. In short time intervals, the cost of switching from the external to the internal time processing is greater than checking the external clock frequently. As a result, in short durations, people mostly rely on the external clock to complete a time-based PM task, and time estimation does not play a critical role in performing the PM task. However, in long time intervals, carrying out the passage of time externally as an exclusive time monitoring strategy is demanding. As a result, it may be that individuals shift from the external to internal time processing periodically to gain the most possible success in long time-based PM tasks. Thus, future research could evaluate whether time estimation plays a more prominent role in long time intervals compared to short ones. This final area of research has strong potential for advancing our theoretical understanding of time-based PM and developing applied interventions to improve performance on these tasks outside of the laboratory.

## Data Availability

Not applicable.

## Footnotes

1 As noted by Smith (2003), some earlier work recognized the dual-task nature of PM tasks, including some studies investigating time-based tasks (e.g., d’Ydewalle, 1995, d’Ydewalle et al., 1999; Park et al. 1997), but earlier work did not emphasize the importance of investigating how including the PM task might influence ongoing task performance.

2 Specific examples of the tasks used in the MIST, which is a copyrighted clinical test, can be found in the open access manuscript available through the National Institutes of Health’s National Library of Medicine at the following link: https://pmc.ncbi.nlm.nih.gov/articles/PMC3057368/.

3 Figure 2 provides a visual representation of the individual study effect sizes (shown as squares) along with their confidence intervals (represented by horizontal lines). The overall effect size (represented as a diamond at the bottom of the plot) indicates the combined effect across all studies, with its width representing the confidence interval for the pooled effect. Points to the right of zero indicate a better PM performance in time-based tasks with shorter time intervals.

4 A funnel plot is a scatter plot commonly used in meta-analyses to evaluate publication bias. In the absence of bias, the plot appears as a symmetrical inverted funnel, with larger, more precise studies concentrated near the top and smaller, less precise studies spread out toward the bottom. Any asymmetry in the plot may indicate the presence of publication bias or other factors affecting the study results.

5 Two additional related studies are not included in the meta-analysis because they did not examine the correlations between time-based PM and time perception. Gan and Guo (2019) conducted two experiments to investigate the potential improvement of time-perception and time-based PM performance through time-based PM and time-perception training, respectively, and found that time-estimation training also enhanced time-based PM performance and clock checking. Waldrum and McDaniel (2016) examined the impact of participants’ time estimation on their performance in a time-based PM task and found overall that prior experience in time-estimation tasks affects time-based PM performance.

6 A reviewer suggested that this proposal would perhaps lead to findings of a more of linear function of clock checks over the time interval rather than the J-shaped curve of clock checks that some studies have found. While most studies indeed report a J-shaped pattern of clock-checking behavior, it is worth noting that some studies have documented deviations from this pattern (see Ceci & Bronfenbrenner, 1985, for an example). This suggests that clock-checking behavior and the strategies individuals employ to decide when to check the clock remain complex and not fully understood. We mention the possibility of subdividing longer intervals into shorter intervals as one potential strategy individuals might use and acknowledge a variety of strategies could underlie clock-checking behavior in time-based prospective memory tasks and the selection of strategies as a function of delay is an area in need of further investigation. At the same time, the J-shaped pattern many studies find is not inconsistent with our subdivision proposal. An individual may complete several initial checks to calibrate their monitoring process and then only periodically check the clock to see if they are in the next subinterval, with an increase in frequency of checking within a given subinterval for subintervals closer to the target, producing a more J-shaped pattern.

## Code availability

R Code.
